# Horizontal Violence Toward Emergency Medicine Residents: Gender as a Risk Factor

**DOI:** 10.5811/westjem.2022.6.55485

**Published:** 2022-08-19

**Authors:** Ashley A. Jacobson, James E. Colletti, Neha P. Raukar

**Affiliations:** * Mayo Clinic Health System, Department of Emergency Medicine, Eau Claire, Wisconsin; † Mayo Clinic, Department of Emergency Medicine, Rochester, Minnesota

## Abstract

**Introduction:**

Horizontal violence (HV) is defined as “persistent exposure to interpersonal aggression and mistreatment from colleagues.” Our objective in this pilot, single-site study was to identify sources of HV toward emergency medicine (EM) residents, using the Negative Acts Questionnaire-Revised (NAQ-R).

**Methods:**

In this investigation we used a descriptive cross-sectional survey design to categorize HV. All voluntary participants were residents in an Accreditation Council for Graduate Medical Education-approved, three-year academic EM residency. Data were collected via electronic survey and occurred six months into an academic year. We collected demographic information and responses to the NAQ-R in 2020. Horizontal violence is subdivided into three categories: work-related; person-related; and physical intimidation. Emergency medicine residents answered questions as they related to their interactions with residents and support staff, which included nursing.

**Results:**

A total of 23 of 26 residents responded (89%). Participants were 56% women, 78% white, 11% Hispanic, and 89% heterosexual. Participant clinical year was 39% first-, 39% second-, and 22% third-year residents. Women reported a higher frequency of HV compared to men (1.3 vs 1.1, P =.01). By category, women indicated higher incidence of work-related violence from other residents (P = .05) and staff (P =.02). There was no difference in reported frequency of violence for interns compared to senior residents.

**Conclusion:**

Our pilot study demonstrated horizontal violence toward EM residents exists and is more prevalent in women.

## INTRODUCTION

The hierarchical structure of education in healthcare is a known risk factor for workplace bullying.^1^–^7^ Workplace bullying is defined as “harassing, offending, socially excluding someone, or negatively affecting someone’s work…occur[ing] repeatedly and regularly (weekly) and over a period of time (eg, about six months).”^8^

Horizontal violence (HV), “persistent exposure to interpersonal aggression and mistreatment from colleagues,”^9^ has predominately been researched within the nursing field^10^–^11^ with interest in resident-directed HV only recently gaining momentum.^7^,^12^–^13^ Resident-directed HV is comprised of staff-to-resident and resident-to-resident bullying. This study focused on HV and did not evaluate vertical violence (attending-to-resident bullying). The general surveys globally used to assess attending and resident physician workplace bullying are the Negative Acts Questionnaire-Revised (NAQ-R),^4^–^6^,^12^–^14^ a bullying scale predominantly used within the United Kingdom,^1^,^3^ and various single-site questionnaires.^15^–^17^

Worldwide, workplace bullying of residents has been identified.^7^ In the US, Daugherty et al^15^ found that after intern year, 62.9% of residents had experienced mistreatment from any source (eg, medical student, resident, attending, nurse, patient). A subsequent study elucidated that 66% of US trainees across all years and specialties experienced at least one type of bullying behavior from either an attending, nurse, patient, peer, consultant, or ancillary staff, with female, non-white residents reporting higher frequency of these episodes.^1^ Workplace bullying of resident physicians is associated with increased psychological distress, increased depressive symptoms, and a positive post-traumatic stress disorder screening.^3^,^18^–^19^

Overall, there is a paucity of data regarding HV specifically and its adverse effects on residents, especially residents in EM – a specialty that depends on frequent interactions with staff and residents from different services. In this pilot study we hypothesized that women residents in their first year of residency training would experience more HV, specifically from other residents and support staff, as measured by a tailored healthcare version of the 22-item NAQ-R.

## METHODS

This pilot study used a descriptive cross-sectional design to determine HV specifically within an EM residency program. All participants were residents within an academic, Level I trauma center in the United States. A voluntary, electronic version of the NAQ-R, that has been used in other healthcare residency settings,^12^–^13^ was disseminated. Data were collected anonymously in 2020, six months into the resident’s current year of training. Data collected included demographic information and responses to the NAQ-R. All data were blinded prior to analysis to decrease the risk associated with surveying a vulnerable population. This study was deemed exempt by the institutional review board. There was no external funding to support this project.

We chose the 22-item NAQ-R as the survey instrument as it is considered the gold standard worldwide (Appendix A). The NAQ-R assesses bullying related to work, personhood, and physical intimidation. Work-related HV questions focus on withholding information, ignoring orders, and excessive monitoring. Person-related HV questions focus on humiliation, gossip, ridicule, and insults. Physical intimidation HV questions focus on shouting, finger-pointing, and physical violence.^9^,^12^–^13^ These questions have been previously tailored to represent the healthcare environment and have been previously validated within general surgery and obstetrics and gynecology residency populations.^13^

Bullying is evaluated in two different ways within the NAQ-R. The NAQ-R originally used an operational definition in 2009; in 2012, the authors reanalyzed the original data to create a cut-off score definition. This was done to improve analysis related to prevalence of workplace bullying. Current literature primarily focuses on the quantitative definition. The operational definition defines bullying as experiencing a negative act once per week during the prior six months; to determine these criteria, survey item responses of “weekly” or any response of “daily” corresponded to each operationalized definition of bullying. The quantitative definition of bullying takes the total score of the 22-item NAQ-R (maximum score 110 if answered “daily” to all questions), and those with total scores greater than 33 are classified as bullying. 4,9,20–21

Residents were asked to complete the 22 questions as they related specifically to other residents, including co-residents, off-service residents, and consulting service residents. They then answered the 22 questions as they related to support staff, including nurses, respiratory therapists, lab technicians, personal care assistants, care team assistants, and finance representatives. We summarized the data with medians and interquartile ranges or with frequency counts and percentages, as applicable. Survey items were presented to respondents using a descriptive Likert scale and were subsequently coded from 1 to 5 with 1 (never), 2 (now and then), 3 (monthly), 4 (weekly), and 5 (daily). A total response score was computed by adding the responses across all 22 survey items. Additionally, we further grouped the survey items into categories of work-related, person-related, and physical intimidation. Data analysis was completed using R Core Team (2019) software (R Foundation for Statistical Computing, Vienna, Austria). Gender and postgraduate year responses to event-frequency questions were performed using Wilcoxon rank sum tests. To avoid issues of multiple comparisons, all *P*-values were adjusted using the false discovery rate correction. All tests were two-sided, and *P*-values less than 0.05 were considered significant.

## RESULTS

A total of 23 of 26 residents completed the questionnaire for a response rate of 88.5%. The table summarizes demographic data. Five respondents only completed the resident portion of the questionnaire. These data are included within the resident analysis making the resident data analysis out of 23 participants; the support staff data analysis included 18 total participants.

From the operational NAQ-R definition of bullying, 13.0% of respondents (3/23) reported being bullied by residents, and 11.1% of respondents (2/18) indicated being bullied by support staff once a week. When the quantitative bullying score (>33 points) was used, 17.4% of respondents were bullied by residents (4/23) and 27.8% were bullied by support staff (5/18); there was no significant difference between support staff and resident bullying (*P* =.471). Overall, women reported a higher frequency of HV compared to men (1.3 vs 1.1, P =.01). When subdividing HV into the three categories of work-related violence, person-related violence, and physical intimidation categories, women indicated a higher incidence of work-related violence, both from residents (*P*= .05) and from support staff (*P* =.02) as viewed in the Figure. There was no difference in reported violence between clinical years.

## DISCUSSION

The literature has focused on HV experienced by nurses; this study highlights that residents also experience HV. The HV that was reported during the first six months of the clinical year demonstrates more EM women residents experience overall HV from both cohorts – residents and support staff. Overall, there is statistical significance between gender, specifically in work-related HV. This specific subset of HV consists of ignoring orders/withholding of information. Men, on the other hand, seem to have a consistent experience with notably less HV than women. Clinical year did not affect HV reported.

Our findings are in line with prior studies that found residents experience more work-related HV overall and that women residents experience more bullying.^1^,^7^,^12^–^13^ Future work should expand this pilot study to include a more heterogeneous population of EM residents across multiple EM residency programs to evaluate the role of race, ethnicity, and sexual orientation to help inform the creation of future interventions aimed at reducing HV. Further studies will be needed to determine what type of interventions need to be implemented.

## LIMITATIONS

Limitations to this pilot study include the small sample size of 26 possible residents, which limited the ability to perform a robust statistical analysis. As this was a self-reported questionnaire, the data may be influenced by recall bias. Age was not included in the demographic portion of the questionnaire, which may be an important variable to consider as well. Unfortunately, not everyone completed all the demographic questions.

## CONCLUSION

The ED is a complex work environment with high-acuity patients presenting in a time-sensitive manner with frequent communication between sub-specialties and admitting services. The addition of residents adds to the complexity of patient care for learners and staff. It is noteworthy that even with a small sample size and homogeneous resident population, gender is a potential factor as to who experiences horizontal violence and from which sources. Overall, this study highlights an area of opportunity to improve the educational experience of residents. Recognizing that gender may be an indicator for HV during resident training is an important first step to ultimately creating a safer learning environment.

## Supplementary Information



## Figures and Tables

**Figure:**
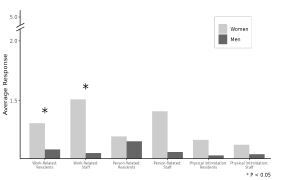
Horizontal violence (HV) presented as average survey response and broken down by gender and the three subcategories of HV: work-related, person-related, and physical intimidation. The self-reported frequency of violence is scored from 1 (never) to 5 (daily). Women experienced a higher incidence of work-related violence, both from other residents (P =.05) and from support staff members (P =.02). This was statistically significant in comparison to men.

**Table:** Demographics of residents who participated in survey on frequency of horizontal violence.

Demographics	Number of Respondents (N = 18^a^)
Gender	
Women	10 (56%)
Men	8 (44%)
Postgraduate Year (PGY)	
PGY 1	7 (39%)
PGY 2	7 (39%)
PGY 3	4 (22%)
Race	
American Indian or Alaska Native	0 (0%)
Asian	0 (0%)
Black or African American	0 (0%)
Native Hawaiian or other Pacific Islander	0 (0%)
White	14 (78%)
Other/Did not disclose	4 (22%)
Ethnicity	
Hispanic or Latino	2 (11%)
Not Hispanic or Latino	15 (83%)
Did not disclose	1 (6%)
Sexual orientation	
Bisexual	1 (6%)
Heterosexual or straight	16 (89%)
Lesbian or gay	0 (0%)
Did not disclose	1 (6%)

a 5 Respondents did not disclose any demographic information.

## References

[1] Chadaga AR, Villines D, Krikorian A (2016). Bullying in the American graduate medical education system: a national cross-sectional survey. PLoS One.

[2] Karim S, Duchcherer M (2014). Intimidation and harassment in residency: a review of the literature and results of the 2012 Canadian Association of Interns and Residents National Survey. Can Med Educ J.

[3] Quine L (2003). Workplace bullying, psychological distress, and job satisfaction in junior doctors. Camb Q Healthc Ethics.

[4] Rahm GB, Rystedt I, Wilde-Larsson B (2019). Workplace bullying among healthcare professionals in Sweden: a descriptive study. Scand J Caring Sci.

[5] Rouse LP, Gallagher-Garza S, Gebhard RE (2016). Workplace bullying among family physicians: a gender focused study. J Womens Health (Larchmt).

[6] Westbrook J, Sunderland N, Li L (2021). The prevalence and impact of unprofessional behaviour among hospital workers: a survey in seven Australian hospitals. Med J Aust.

[7] Samsudin EZ, Isahak M, Rampal S (2018). The prevalence, risk factors and outcomes of workplace bullying among junior doctors: a systematic review. Eur J Work Organ.

[8] Matthiesen SB, Einarsen S (2010). Bullying in the workplace: definition, prevalence, antecedents and consequences. Int J Organ Theory Behav.

[9] Einarsen S, Hoel H, Notelaers G (2009). Measuring exposure to bullying and harassment at work: validity, factor structure and psychometric properties of the Negative Acts Questionnaire-Revised. Work Stress.

[10] Bambi S, Foà C, De Felippis C (2018). Workplace incivility, lateral violence and bullying among nurses. A review about their prevalence and related factors. Acta Biomed.

[11] Serafin L, Sak-Dankosky N, Czarkowska-Pączek B (2020). Bullying in nursing evaluated by the Negative Acts Questionnaire-Revised: A systematic review and meta-analysis. J Adv Nurs.

[12] Chowdhury ML, Husainat MM, Suson KD (2019). Workplace bullying of urology residents: implications for the patient and provider. Urology.

[13] Schlitzkus LL, Vogt KN, Sullivan ME (2014). Workplace bullying of general surgery residents by nurses. J Surg Educ.

[14] Ling M, Young CJ, Shepherd HL (2016). Workplace bullying in surgery. World J Surg.

[15] Daugherty SR, Baldwin DWC, Rowley BD (1998). Learning, satisfaction, and mistreatment during medical internship. JAMA.

[16] Volz N, Fringer R, Walters B (2017). Prevalence of horizontal violence among emergency attending physicians, residents, and physician assistants. West J Emerg Med.

[17] Wolfman DJ, Parikh JR (2019). Resident bullying in diagnostic radiology. Clin Imaging.

[18] Loerbroks A, Weigl M, Li J (2015). Workplace bullying and depressive symptoms: a prospective study among junior physicians in Germany. J Psychosom Res.

[19] Jackson T, Provencio A, Bentley-Kumar K (2017). PTSD and surgical residents: Everybody hurts… sometimes. Am J Surg.

[20] Mikkelsen EG, Einarsen S (2001). Bullying in Danish work-life: prevalence and health correlates. Eur J Work Organ.

[21] Notelaers G, Einarsen S (2013). The world turns at 33 and 45: defining simple cutoff scores for the NEGATIVE Acts Questionnaire–revised in a representative sample. Eur J Work Organ.

