# Supplementary material for: Horizontal Violence Toward Emergency Medicine Residents: Gender as a Risk Factor
Source: West J Emerg Med. 2022 Aug 19;23(5):633–6. doi: 10.5811/westjem.2022.6.55485 (PMC9541973; doi:10.5811/westjem.2022.6.55485)
Supplement: Supplementary file 1 [file wjem-23-633-s001.pdf]

Supplemental material attached below.

Q1 **Assessment of Workplace Environment for Residents within the Emergency Department**

During the past 6 months of your residency, has a *resident* (co-resident, off-service resident, consulting service resident) or NP/PA ever:

	Never (1)	Now and then (2)	Monthl y (3)	Weekly (4)	Daily (5)
Withheld information that affected your performance? (1)					
Humiliated or ridiculed you in connection with your work? (2)					
Asked you to do work below your level of competence such as asking a chief to do intern level work? (3)					
Made you or expected you to perform trivial or unpleasant tasks in addition to your normal responsibilities such as scut work, changing bedpans, etc.? (4)					
Spread gossip and rumors about you? (5)					
Ignored or excluded you? (6)					
Insulted or made offensive remarks about you, your attitude, or your private life? (7)					
Shouted at you or made you the target of their spontaneous anger? (8)					
Demonstrated intimidating behaviors towards you such as pointing their finger at you, invading your personal space, shoving you, or blocking your view or way? (9)					
Made hints or signals that you should quit your job? (10)					
Repeatedly reminded you of your errors or mistakes? (11)					

Q2 **During the past 6 months of your residency, has a *resident* (co-resident, off-service resident, consulting service resident) or NP/PA ever**:

	Never (1)	Now and then (2)	Monthl y (3)	Weekly (4)	Daily (5)
Ignored you when you approached them or have you ever faced a hostile reaction when you approached them? (1)					
Persistently criticized you about your errors or mistakes? (2)					
Ignored your recommendations or orders? (3)					
Made you the butt of a practical joke? (4)					
Made a request of you with an unreasonable deadline? (5)					
Made allegations against you? (6)					
Excessively monitored your work? (7)					
Pressured you not to report a near miss incident or critical patient error? (8)					
Excessively teased you or been excessively sarcastic toward you? (9)					
Deliberately contributed to an unmanageable workload? (10)					
Threatened violence against you or physically abused you? (11)					

Q3

During the past 6 months of your residency, has a *resident* (co-resident, off-service resident, consulting service resident) or NP/PA ever:

	Check here if you witnessed any FEMALE residents subjected to this behavior (1)	Check here if you witnessed any MALE residents subjected to this behavior (2)
Withheld information that affected the female/male resident's performance? (1)		
Humiliated or ridiculed the female/male resident in connection with their work? (2)		
Asked the female/male resident to do work below their level of competence such as asking a chief to do intern level work? (3)		
Made the female/male resident or expected them to perform trivial or unpleasant tasks in addition to their normal responsibilities such as scut work, changing bedpans, etc.? (4)		
Spread gossip and rumors about the female/male resident? (5)		
Ignored or excluded the female/male resident? (6)		
Insulted or made offensive remarks about the female/male resident, their attitude, or their private life? (7)		
Shouted at the female/male resident or made them the target of spontaneous anger? (8)		
Demonstrated intimidating behaviors towards female/male resident such as pointing a finger at them, invading their personal space, shoving them, or blocking their view or way? (9)		
Made hints or signals that the female/male resident should quit their job? (10)		
Repeatedly reminded the female/male resident of their errors or mistakes? (11)		

Q4

During the past 6 months of your residency, has a *resident* (co-resident, off-service resident, consulting service resident) or NP/PA ever:

	Check here if you witnessed any FEMALE residents subjected to this behavior (1)	Check here if you witnessed any MALE residents subjected to this behavior (2)
Ignored or had a hostile reaction when the female/male resident approached them? (1)		
Persistently criticized the female/male resident about their errors or mistakes? (2)		
Ignored the female/male resident's recommendations or orders? (3)		
Made the female/male resident the butt of a practical joke? (4)		
Made a request of the female/male resident with an unreasonable deadline? (5)		
Made allegations against the female/male resident? (6)		
Excessively monitored the female/male resident's work? (7)		
Pressured the female/male resident not to report a near miss incident or critical patient error? (8)		
Excessively teased the female/male resident or been excessively sarcastic toward them? (9)		
Deliberately contributed to an unmanageable workload? (10)		
Threatened violence against the female/male resident or physically abused them? (11)		

Q5 **During the past 6 months of your residency, has a <u>support staff member</u> (nurse, pharmacist, respiratory therapist, lab technician, personal care assistant, care team assistant, or finance representative) ever:**

	Never (1)	Now and then (2)	Monthly (3)	Weekly (4)	Daily (5)
Withheld information that affected your performance? (1)					
Humiliated or ridiculed you in connection with your work? (2)					
Asked you to do work below your level of competence such as asking a chief to do intern level work? (3)					
Made you or expected you to perform trivial or unpleasant tasks in addition to your normal responsibilities such as scut work, changing bedpans, etc.? (4)					
Spread gossip and rumors about you? (5)					
Ignored or excluded you? (6)					
Insulted or made offensive remarks about you, your attitude, or your private life? (7)					
Shouted at you or made you the target of their spontaneous anger? (8)					
Demonstrated intimidating behaviors towards you such as pointing their finger at you, invading your personal space, shoving you, or blocking your view or way? (9)					
Made hints or signals that you should quit your job? (10)					
Repeatedly reminded you of your errors or mistakes? (11)					

Q6 **During the past 6 months of your residency, has a <u>support staff member</u> (nurse, pharmacist, respiratory therapist, lab technician, personal care assistant, care team assistant, or finance representative) ever**:

	Never (1)	Now and then (2)	Monthly (3)	Weekly (4)	Daily (5)
Ignored you when you approached them or have you ever faced a hostile reaction when you approached them? (1)					
Persistently criticized you about your errors or mistakes? (2)					
Ignored your recommendations or orders? (3)					
Made you the butt of a practical joke? (4)					
Made a request of you with an unreasonable deadline? (5)					
Made allegations against you? (6)					
Excessively monitored your work? (7)					
Pressured you not to report a near miss incident or critical patient error? (8)					
Excessively teased you or been excessively sarcastic toward you? (9)					
Deliberately contributed to an unmanageable workload? (10)					
Threatened violence against you or physically abused you? (11)					

Q7 **During the past 6 months of your residency, has a <u>support staff member</u> (nurse, pharmacist, respiratory therapist, lab technician, personal care assistant, care team assistant, or finance representative) ever:**

	Check here if you witnessed any FEMALE residents subjected to this behavior (1)	Check here if you witnessed any MALE residents subjected to this behavior (2)
Withheld information that affected the female/male resident's performance? (1)		
Humiliated or ridiculed the female/male resident in connection with their work? (2)		
Asked the female/male resident to do work below their level of competence such as asking a chief to do intern level work? (3)		
Made the female/male resident or expected them to perform trivial or unpleasant tasks in addition to their normal responsibilities such as scut work, changing bedpans, etc.? (4)		
Spread gossip and rumors about the female/ male resident? (5)		
Ignored or excluded the female/male resident? (6)		
Insulted or made offensive remarks about the female/male resident, their attitude, or their private life? (7)		
Shouted at the female/male resident or made them the target of spontaneous anger? (8)		
Demonstrated intimidating behaviors towards female/male resident such as pointing a finger at them, invading their personal space, shoving them, or blocking their view or way? (9)		
Made hints or signals that the female/male resident should quit their job? (10)		
Repeatedly reminded the female/male resident of their errors or mistakes? (11)		

Q8 **During the past 6 months of your residency, has a <u>support staff member</u> (nurse, pharmacist, respiratory therapist, lab technician, personal care assistant, care team assistant, or finance representative) ever:**

	Check here if you witnessed any FEMALE residents subjected to this behavior (1)	Check here if you witnessed any MALE residents subjected to this behavior (2)
Ignored or had a hostile reaction when the female/male resident approached them? (1)		
Persistently criticized the female/male resident about their errors or mistakes? (2)		
Ignored the female/male resident's recommendations or orders? (3)		
Made the female/male resident the butt of a practical joke? (4)		
Made a request of the female/male resident with an unreasonable deadline? (5)		
Made allegations against the female/male resident? (6)		
Excessively monitored the female/male resident's work? (7)		
Pressured the female/male resident not to report a near miss incident or critical patient error? (8)		
Excessively teased the female/male resident or been excessively sarcastic toward them? (9)		
Deliberately contributed to an unmanageable workload? (10)		
Threatened violence against the female/ male resident or physically abused them? (11)		

Q9 **What sex were you assigned at birth?**

- ◯ Female (1)
- ◯ Male (2)
- ◯ Intersex (3)

Q10 **What is your current gender identity?**

- ◯ Female (1)
- ◯ Male (2)
- ◯ Transgender female (male-to-female) (3)
- ◯ Transgender male (female-to-male) (4)
- ◯ Neither exclusively male nor female, non-binary or genderqueer (5)
- ◯ Other (6)
- ◯ Choose not to disclose (7)

Q11 **Do you think of yourself as?**

- ◯ Asexual (1)
- ◯ Bisexual (2)
- ◯ Heterosexual or straight (3)
- ◯ Homosexual, lesbian, or gay (4)
- ◯ Pansexual (5)
- ◯ Queer questioning (6)
- ◯ Something else (7)
- ◯ Choose not to disclose (8)

Q12 **What is your ethnicity?**

- ◯ Hispanic or Latino (1)
- ◯ Not Hispanic or Latino (2)

Q13 **What is your race?** (Mark all that apply.)

- ☐ American Indian or Alaska Native (1)
- ☐ Asian (2)
- ☐ Black or African American (3)
- ☐ Native Hawaiian or Other Pacific Islander (4)
- ☐ White (5)

Q14 **What clinical year are you?**

- ○ PGY 1 (1)
- ○ PGY 2 (2)
- ○ PGY 3 (3)
